# Supplementary material for: Could Children’s Myopization Have Been Avoided during the Pandemic Confinement? The Conjunctival Ultraviolet Autofluorescence (CUVAF) Biomarker as an Answer
Source: Biomedicines. 2024 Feb 1;12(2):347. doi: 10.3390/biomedicines12020347 (PMC10886979; doi:10.3390/biomedicines12020347)
Supplement: Supplementary file 1 [file biomedicines-12-00347-s001.zip › biomedicines-2840647-supplementary.pdf]

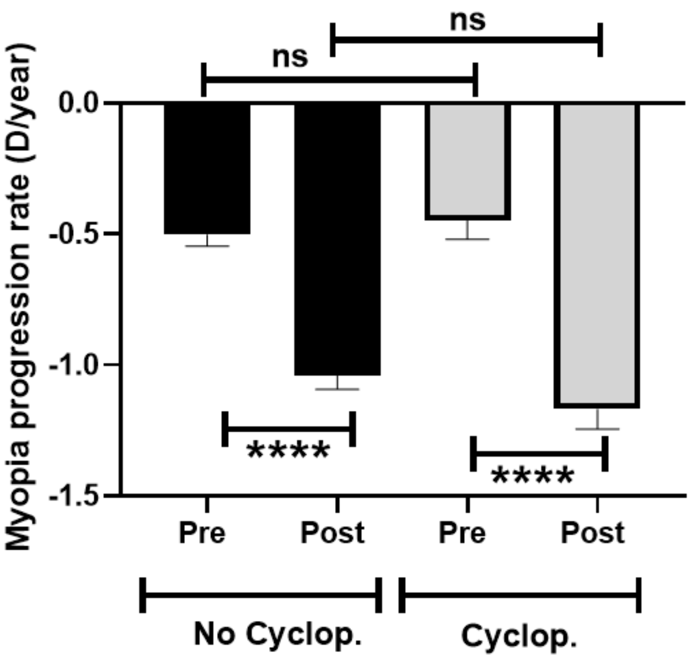

Figure S1. Cycloplegia analysis. Myopia progression rate in children measured with (clinic patients, n=63) and without cycloplegia (optical shops, n=139). ns: non-significant. Significance p < 0.05. **** p < 0.0001.

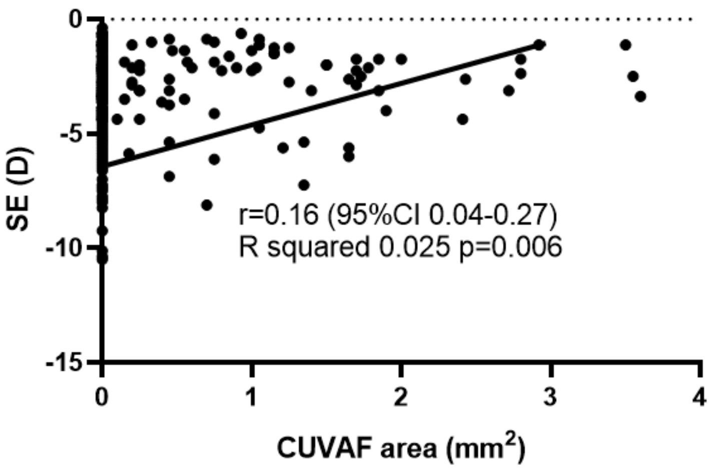

Figure S2. Correlation of Spherical Equivalent and CUVAF area in all the analyzed patients.
